# L-cysteine ethyl ester prevents and reverses acquired physical dependence on morphine in male Sprague Dawley rats

**DOI:** 10.3389/fphar.2023.1303207

**Published:** 2023-12-04

**Authors:** James N. Bates, Paulina M. Getsy, Gregory A. Coffee, Santhosh M. Baby, Peter M. MacFarlane, Yee-Hsee Hsieh, Zackery T. Knauss, Jason A. Bubier, Devin Mueller, Stephen J. Lewis

**Affiliations:** ^1^ Department of Anesthesiology, University of Iowa Hospitals and Clinics, Iowa City, IA, United States; ^2^ Department of Pediatrics, Case Western Reserve University, Cleveland, OH, United States; ^3^ Section of Biology, Galleon Pharmaceuticals, Inc., Horsham, PA, United States; ^4^ Division of Pulmonary, Critical Care and Sleep Medicine, Case Western Reserve University, Cleveland, OH, United States; ^5^ Department of Biological Sciences, Kent State University, Kent, OH, United States; ^6^ Jackson Laboratories, Bar Harbor, ME, United States; ^7^ Department of Pharmacology, Case Western Reserve University, Cleveland, OH, United States; ^8^ Functional Electrical Stimulation Center, Case Western Reserve University, Cleveland, OH, United States

**Keywords:** opioids, morphine, naloxone, physical dependence, withdrawal, L-cysteine, L-cysteine ethyl ester, rats

## Abstract

The molecular mechanisms underlying the acquisition of addiction/dependence on morphine may result from the ability of the opioid to diminish the transport of L-cysteine into neurons via inhibition of excitatory amino acid transporter 3 (EAA3). The objective of this study was to determine whether the co-administration of the cell-penetrant L-thiol ester, L-cysteine ethyl ester (L-CYSee), would reduce physical dependence on morphine in male Sprague Dawley rats. Injection of the opioid-receptor antagonist, naloxone HCl (NLX; 1.5 mg/kg, IP), elicited pronounced withdrawal phenomena in rats which received a subcutaneous depot of morphine (150 mg/kg) for 36 h and were receiving a continuous infusion of saline (20 μL/h, IV) via osmotic minipumps for the same 36 h period. The withdrawal phenomena included wet-dog shakes, jumping, rearing, fore-paw licking, 360° circling, writhing, apneas, cardiovascular (pressor and tachycardia) responses, hypothermia, and body weight loss. NLX elicited substantially reduced withdrawal syndrome in rats that received an infusion of L-CYSee (20.8 μmol/kg/h, IV) for 36 h. NLX precipitated a marked withdrawal syndrome in rats that had received subcutaneous depots of morphine (150 mg/kg) for 48 h) and a co-infusion of vehicle. However, the NLX-precipitated withdrawal signs were markedly reduced in morphine (150 mg/kg for 48 h)-treated rats that began receiving an infusion of L-CYSee (20.8 μmol/kg/h, IV) at 36 h. In similar studies to those described previously, neither L-cysteine nor L-serine ethyl ester (both at 20.8 μmol/kg/h, IV) mimicked the effects of L-CYSee. This study demonstrates that 1) L-CYSee attenuates the development of physical dependence on morphine in male rats and 2) prior administration of L-CYSee reverses morphine dependence, most likely by intracellular actions within the brain. The lack of the effect of L-serine ethyl ester (oxygen atom instead of sulfur atom) strongly implicates thiol biochemistry in the efficacy of L-CYSee. Accordingly, L-CYSee and analogs may be a novel class of therapeutics that ameliorate the development of physical dependence on opioids in humans.

## Introduction

There are numerous problems faced by clinicians treating patients with opioid use disorder (OUD) and other substance use disorders (SUDs) (McCarberg, 2011; Heberlein et al., 2012; Garcia-Portilla et al., 2014; Hah et al., 2017; Dydyk et al., 2022; 2023; Matson et al., 2022; Horn et al., 2023). With regard to managing pain in the general population, the key issues are how to provide μ-opioid receptor (μ-OR) agonist (e.g., fentanyl)-induced analgesia without eliciting 1) euphoria, 2) physical dependence and/or psychological addiction, 3) hyperalgesia, and 4) any combination of the aforementioned conditions (Benyamin et al., 2008; Volkow et al., 2018; Mercadante et al., 2019; Manhapra, 2022; Preux et al., 2022; Balanaser et al., 2023). With respect to treating moderate-to-severe OUD (DSM-5 terminology for opioid addiction in 10%–20% of people who have liability for SUD), the key issues are 1) how to manage the often severe opioid withdrawal (all current strategies to manage opioid withdrawal are inadequate); 2) how to manage patients who have gone through withdrawal and need medication to block euphoria and/or physical dependence, with the three medications available, naltrexone, buprenorphine, and methadone, all having strengths, but major weaknesses; and 3) how to avoid opioid euphoria and/or physical dependence in patients with moderate-to-severe OUD who currently are sober, but require μ-OR agonist analgesia (Humphreys et al., 2022; Jiménez-Fernández et al., 2022; Torres-Lockhart et al., 2022; Alvarez-Perez et al., 2023; Biancuzzi et al., 2023; Frankeberger et al., 2023). With respect to effectively treating SUD involving opioids and other substances (e.g., alcohol, cannabinoids, benzodiazepines, and psycho-stimulants), in 10%–20% of the population prone to SUD, the important issue is how to provide (yet to be developed) effective therapeutics that will attenuate/block dopamine surge-mediated euphoria of each of these families of brain-reward drugs as an adjunct to treating addictive diseases (Chartoff and Connery, 2014; Stephan and Parsa, 2016; Horsfall and Sprague, 2017; Bechara et al., 2019; Serafini and Zachariou, 2019). With respect to making opioid analgesics safer, we lack drugs that effectively modulate the actions of opioids to improve their analgesic profile. The therapeutics should 1) not interfere with or promote opioid analgesia, 2) prevent the acquisition of physical dependence and psychological addiction to opioids, 3) block opioid-induced respiratory depression (OIRD), or 4) stop the development of hyperalgesia (Benyamin et al., 2008; Morgan and Christie, 2011; Volkow et al., 2018; Mercadante et al., 2019; Manhapra, 2022; Preux et al., 2022; Balanaser et al., 2023).

Trivedi et al. (2014) reported that morphine-induced dependence/addiction may involve redox-based changes in global DNA methylation and retrotransposon transcription via the blockade of excitatory amino acid transporter type 3 (EAA3; also known as EAAC1)-dependent uptake of the amino acid, L-cysteine, into brain neurons. Some of the temporal steps described by Trivedi et al. (2014) (see Figure 5 of Trivedi et al., 2014) and others (Lin et al., 2001; Ikemoto et al., 2002; Mao et al., 2002; Xu et al., 2003; Xu et al., 2006; Christie, 2008; Yang et al., 2008; Wang et al., 2009; Daijo et al., 2011; Gutowicz et al., 2011; Liu et al., 2011; Maze and Nestler, 2011; Lim et al., 2012; Sun et al., 2012; Browne et al., 2020) are as follows: 1) morphine blockade of L-cysteine uptake into neurons by G protein-dependent inhibition of EAA3 activity, 2) resulting decreases in intracellular levels of L-cysteine, L-glutathione, and the methylation index, namely, S-adenosyl-methionine/S-adenosyl-homocysteine (SAM/SAH ratio), 3) decreases in the methylation status of global CpG (regions of DNA where a cytosine nucleotide is followed by a guanine nucleotide in the linear sequence of bases along its 5′ → 3′ direction) and in CpG methylation of long interspersed nuclear element-1 (LINE-1) retrotransposon regulatory regions, and 4) activation of transcription of previously silenced LINE-1 genes. Thus, we hypothesized that co-administration of cell-permeant versions of L-cysteine, such as L-cysteine ethyl ester (L-CYSee) (Goto et al., 1983; Hisadome et al., 1986a; Hisadome et al., 1986b; Hisadome et al., 1988; Servin et al., 1988; Hisadome et al., 1990; Schöneich et al., 1992; Hobbs et al., 1993; Fukui et al., 1994; Ding and Demple, 1998; Galanakis et al., 2004; Mosier-Boss and Lieberman, 2005; Perissinotti et al., 2005; Defonsi Lestard et al., 2013; Mendoza et al., 2013; Arias et al., 2019), may prevent the acquisition of physical dependence on morphine and reverse established dependence on the opioid. Previously, we reported that L-CYSee (Lewis et al., 2022), L-cysteine methyl ester (Getsy et al., 2022a), and other thiolesters and related compounds (Baby et al., 2021; Baby et al., 2021; Gaston et al., 2021; Jenkins et al., 2021; Getsy et al., 2022b; Getsy et al., 2022c; Getsy et al., 2022d; Getsy et al., 2022e; Getsy et al., 2022f) prevent and/or reverse the adverse effects of morphine and fentanyl on ventilatory parameters, arterial blood–gas chemistry (pH, pCO_2_, pO_2_, and sO_2_), and alveolar–arterial gradient (index of alveolar gas exchange in the lungs) in freely-moving rats without compromising opioid-induced analgesia or sedation. We now provide evidence that administration of L-CYSee prevents the acquisition of physical dependence, as measured by markedly fewer withdrawal phenomena in response to administration of the opioid receptor antagonist, naloxone HCl (NLX), in freely-moving male rats, and reverses established dependence. The lack of effect of L-cysteine and L-serine ethyl ester (L-SERee, oxygen atom instead of a sulfur atom as in L-CYSee) in these paradigms suggests that the efficacy of L-CYSee involves its cell penetrability in brain regions vital to the expression of morphine dependence, and points to the vital role of thiol biochemistry in the biological efficacy of L-CYSee.

## Materials and methods

### Permissions, rats, and surgical procedures

All studies were carried out in strict accordance with the NIH Guide for Care and Use of Laboratory Animals (NIH Publication No. 80-23) revised in 1996 and in strict compliance with the ARRIVE (Animal Research: Reporting of *In Vivo* Experiments) guidelines (http://www.nc3rs.org.uk/). All protocols involving the use of rats were approved by the Animal Care and Use Committees of Galleon Pharmaceuticals (PC0022), Case Western Reserve University (2015-0025), and the University of Virginia (3642-09-07). Adult male Sprague Dawley rats of approximately 12 weeks of age at the time of study were purchased from *Harlan Industries* (Madison, WI, United States). The rats were given 5 days to recover from transportation before being subjected to surgeries, as described in this paragraph. (+)-Morphine sulfate was obtained from *Baxter Healthcare* (Deerfield, IL, United States). L-cysteine hydrochloride monohydrate (L-cysteine HCl) powder (C7880, PubChem Substance ID:24892992), L-cysteine ethyl ester hydrochloride (L-CYSee HCl) powder (C121908, PubChem Substance ID: 24892386), and L-serine ethyl ester hydrochloride (L-SERee HCl) powder (223123; PubChem Substance ID:24853367) were divided into 100 mg amounts under N_2_ gas and stored at 4°C. Solutions of L-cysteine HCl, L-CYSee HCl, and L-SERee HCl were dissolved in saline and brought to pH 7.2 with 0.1 M NaOH at room temperature immediately before use. Naloxone hydrochloride dihydrate powder (BP548; PubChem Substance ID: 24278050) was obtained from Sigma-Aldrich (St. Louis, MO, USA) and was dissolved in saline at pH 7.2 with 0.1 M NaOH at room temperature immediately before use. On the day of the study, all arterial and venous catheters were flushed with 0.3 mL of phosphate-buffered saline (0.1 M, pH 7.4) 3–4 h before commencement of the study. All studies were done in a quiet room with a relative humidity of 50% ± 2% and room temperature of 21.3°C ± 0.2°C. Every group described in this study contained nine rats. Other than the described surgeries (see the next section), no rat had a prior history of use in any experimental protocol and was used in only one protocol in the present study. The times at which the surgeries were performed will be described herein. Surgery times from the initiation of anesthesia to final closing of all wounds took approximately a) 20 min for the placement of one venous catheter, b) 25 min for the placement of two venous catheters, c) 30 min for the placement of two intravenous and one arterial catheter, and d) 20 min for intravenous catheterization in which the connected osmotic minipump is placed subcutaneously. Two protocols were as follows: 1) Examine the ability of L-CYSee and L-cysteine (behavioral and cardiorespiratory studies), and L-SERee (behavioral study only) to prevent the acquisition of physical dependence on morphine upon 36 h exposure to the opioid and test compounds. The question addressed whether treatment with L-CYSee, for example, diminishes NLX-precipitated withdrawal phenomena; and 2) examine whether the introduction of L-CYSee or L-cysteine (behavioral and cardiorespiratory studies) or L-SERee (behavioral study only) at 36 h of morphine treatment reverses established physical dependence on morphine, as tested after 48 h of morphine treatment. The question addressed for the second protocol is whether co-treatment for 12 h with L-CYSee, for example, reverses existing physical dependence on morphine, as expressed by markedly diminished NLX-precipitated withdrawal phenomena.

### Protocols to determine the effects of L-CYSee on physical dependency of morphine and prevention of morphine dependence—36-h studies

A. Behavioral studies: At 2 a.m. on the day of surgery, two groups of rats received a jugular vein catheter (PE-10 connected to PE-50) under 2%–3% isoflurane anesthesia (Henderson et al., 2014; Gaston et al., 2021; Getsy et al., 2022f). The jugular vein catheter was connected to a primed ALZET osmotic minipump (Model 2002; ALZA Corporation, CA, United States) positioned at the back of the neck to allow continuous infusion of the vehicle (20 μL/h, IV), L-cysteine (20.8 μmol/kg/h, IV), L-CYSee (20.8 μmol/kg/h, IV), or L-SERee (20.8, μmol/kg/h, IV), as described previously (Jarrott et al., 1987; Lewis et al., 1988a; Jarrott et al., 1988; Lewis et al., 1989). Physical dependence was induced by a slow-release subcutaneous depot of morphine emulsion (150 mg/kg, SC) injected at the left side of the neck, as described in detail by Fennessy and colleagues (Lee and Fennessy, 1970; Laska and Fennessy, 1976; Laska and Fennessy, 1977; Laska and Fennessy, 1978; Lewis et al., 1988b). In brief, morphine base was precipitated from a solution of (+)-morphine sulfate by titrating to pH 9 with 1 mol/L NaOH. After several distilled water washes, a pure base was collected in a filter funnel and dried. Morphine slow-release emulsion was prepared by suspending a weighed amount of base in liquid paraffin and *Arlacel A.* This mixture was then emulsified with an equal volume of normal saline, as initially described by Collier et al. (1972). All wounds were sutured closed, and the rats were returned to their warmed home cages. After 35.5 h of morphine administration, the rats were placed in individual opaque plastic boxes; after 30 min of acclimatization, they received an intraperitoneal (IP) injection of NLX (1.5 mg/kg), and behavioral phenomena were scored for 45 min by at least three scorers. The scored phenomena were as follows: jumping behavior—all four paws of the ground—jumps; wet-dog shakes—whole body shakes as if to shed water from fur; rearing behavior—rearing on hind legs—rears; episodes of fore-paw licking—FPL; circling behavior—complete 360° rotation; writhes—full-body contortion; sneezes—episodes of sneezing—abrupt expulsion of air that often disturbed the fine bedding material.
B. Plethysmography ventilatory studies: Groups of rats were prepared as described previously except that the rats received a second catheter into the jugular vein, as described by Getsy et al. (2022f), to give a bolus injection of NLX. After 35 h, rats were placed in individual whole-body plethysmography chambers (Young et al., 2013; Getsy et al., 2022a; Getsy et al., 2022b; Getsy et al., 2022c; Getsy et al., 2022d; Getsy et al., 2022e; Getsy et al., 2022f), and the free end of the exteriorized venous catheter was connected to a swivel assembly housed in the lid of the plethysmography chamber (Young et al., 2013; Getsy et al., 2022a; Getsy et al., 2022b; Getsy et al., 2022c; Getsy et al., 2022d; Getsy et al., 2022e; Getsy et al., 2022f). After a 60-min acclimatization period, the rats were given an intravenous injection of NLX (1.5 mg/kg). Ventilatory parameters including the frequency of breathing, tidal volume, minute ventilation, and non-eupneic breathing indices were recorded (to be reported elsewhere) with the number of apneas (>1.5 s between breaths) reported here.
C. Cardiovascular studies: Groups of rats were prepared as described previously except that the rats received a second catheter into a jugular vein (Getsy et al., 2022f) to administer NLX and a catheter into a femoral artery to continuously record mean arterial blood pressure (MAP) and heart rate (Kanbar et al., 2010; Davisson et al., 2014; Brognara et al., 2016; Gaston et al., 2020). After 35 h, the rats were placed in individual opaque plastic boxes, and the free end of the exteriorized jugular vein catheter was connected to an injection line to deliver NLX. The free end of the arterial line was connected to tubing attached to a computer-coupled pressure transducer (*Cabe Lab, Inc.*) to record pulsatile arterial blood pressure. After a 60-min acclimatization period, the rats received a bolus injection of NLX (1.5 mg/kg, IV), and MAP and heart rate were recorded continuously for 45 min.
D. Body temperature and body weight studies: Groups of rats without a second jugular catheter were prepared as described in the previous paragraph. After 35 h, the rats were placed in individual opaque plastic boxes, and a thermistor probe, used to record body temperature, was connected to a telethermometer (*Yellow Springs Instruments*) and inserted 5–6 cm into the rectum and taped to the tail (Kregel et al., 1997). The body weights of the rats and their body temperatures were recorded every 15 min during the acclimatization period to establish accurate baseline values. After the 60-min acclimatization period, the rats received an intraperitoneal injection of NLX (1.5 mg/kg), and body temperatures and body weights were recorded every 15 min for 90 min.

### Reversal of morphine dependence—48-h studies

A. Behavioral studies: At 2 p.m. on the day of surgery, two groups of rats received a slow-release subcutaneous depot of morphine emulsion (150 mg/kg, SC) injected at the left side of the neck, as described previously. After 36 h of morphine administration, the rats were anesthetized (2% isoflurane) and received a jugular vein catheter connected to a primed ALZET osmotic minipump positioned at the back of the neck for continuous infusion of the vehicle (20 μL/h, IV), L-cysteine (20.8, μmol/kg/h, IV), L-CYSee (20.8 μmol/kg/h, IV), or L-SERee (20.8, μmol/kg/h, IV), as mentioned previously. All wounds were then sutured closed, and the rats were returned to their warmed home cages. After 11.5h, the rats were placed in individual opaque plastic boxes, and after a 30-min period of acclimatization, the rats received an intraperitoneal injection of NLX (1.5 mg/kg), and behavioral phenomena (as detailed previously) were scored for 45 min by at least three scorers.
B. Plethysmography ventilatory studies: Groups of rats were prepared as described previously, except that the rats received two catheters into the same jugular vein, as described by Getsy et al. (2022f), to allow for the bolus injection of NLX via the catheter not connected to the osmotic minipump. After 47 h, rats were placed in individual whole body plethysmography chambers. The free end of the exteriorized jugular vein catheter was connected to a swivel on the lid of the plethysmography chamber. After 60 min of acclimatization, the rats received a bolus injection of NLX (1.5 mg/kg, IV). Ventilatory parameters and non-eupneic breathing indices were recorded, with the number of apneas (>1.5 s between breaths) reported.
C. Cardiovascular studies: Groups of rats were prepared as previously described, except that the rats received two catheters into the same jugular vein to inject NLX via the catheter not connected to the osmotic minipump, and a catheter into a femoral artery to record MAP and heart rate. After 47 h, the rats were placed in individual opaque plastic boxes, and the free end of the exteriorized jugular vein catheter was connected to an injection line to inject NLX. The free end of the arterial line was connected to tubing attached to a computer-coupled pressure transducer to record pulsatile arterial blood pressure. After a 60-min acclimatization period, the rats received an injection of NLX (1.5 mg/kg, IV), and MAP and heart rate were recorded continuously for 45 min.
D. Body temperature and body weight studies: Groups of rats without a second jugular catheter were prepared, as described previously. After 47 h, the rats were placed in individual opaque plastic boxes, and a thermistor probe, used to record body temperature, was connected to a telethermometer (*Yellow Springs Instruments*) and inserted 5–6 cm into the rectum and taped to the tail. The body weights of the rats and body temperatures were recorded every 15 min during acclimatization to establish baseline values. After the 60-min acclimatization period, the rats received an intraperitoneal injection of NLX (1.5 mg/kg). Body temperature and weights were recorded every 15 min for 90 min.

### Data analyses

**FIGURE 1:**
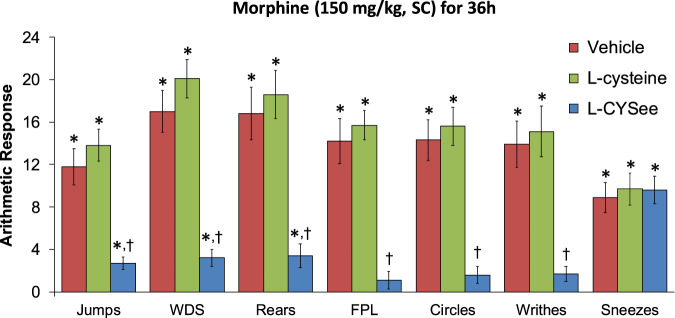
Withdrawal behaviors elicited by a bolus injection of NLX (1.5 mg/kg, IP) in rats treated for 36 h with a subcutaneous depot of morphine (150 mg/kg) along with continuous infusion of the vehicle (saline, 20 μL/h, IV) or L-cysteine (20.8 μmol/kg/h, IV) or L-cysteine ethylester (L-CYSee, 20.8 μmol/kg/h, IV). Withdrawal signs: jumps—all four paws off the floor; WDS-wet-dog shakes; rears—rearing on hind legs; FPL—episodes of fore-paw licking; circles-a 360° rotation; writhes-full-body contortion; sneezes-abrupt expulsion of air. The data are presented as mean ± SEM. There were nine rats in each group. Between-group ANOVA statistics: jumps: F_2,24_ = 18.8, *p* = 0.00001; WDS: F_2,24_ = 29.9, *p* < 0.00001; rears: F_2,24_ = 16.2, *p* = 0.00004; FPL: F_2,24_ = 27.0, *p* < 0.00001; circles: F_2,24_ = 24.1, *p* < 0.00001; writhes: F_2,24_ = 15.4, *p* < 0.00005; sneezes: F_2,24_ = 0.09, *p* = 0.92. *p < 0.05, significant responses from Pre. ^†^ *p* < 0.05, L-CYSee versus vehicle or L-cysteine.

**FIGURE 2:**
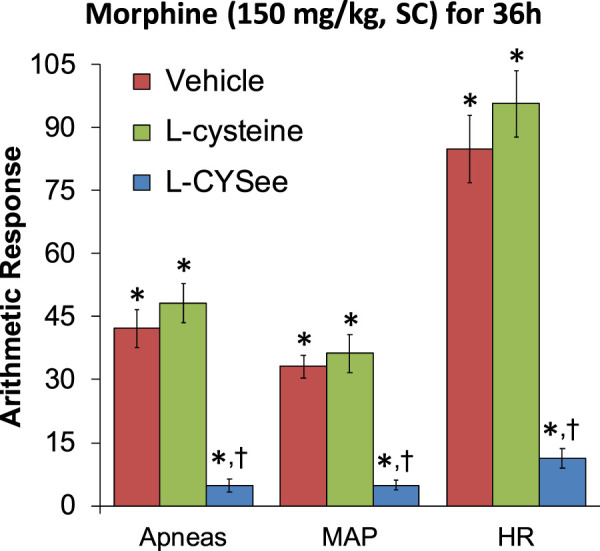
Incidence of apneas (>1.5 s between breaths) and increases in mean arterial blood pressure (MAP, mmHg) and heart rate (HR, beats/min) elicited by a bolus injection of NLX (1.5 mg/kg, IV) in rats treated for 36 h with a subcutaneous depot of morphine (150 mg/kg) along with the continuous infusion of vehicle (saline, 20 μL/h, IV), L-cysteine (20.8 μmol/kg/h, IV), or L-cysteine ethyl ester (L-CYSee, 20.8 μmol/kg/h, IV). The data are presented as mean ± SEM. There were nine rats in each group. Between-group ANOVA statistics: MAP: F_2,24_ = 28.5, *p* < 0.00001; HR: F_2,24_ = 47.8, *p* < 0.00001; apneas: F_2,24_ = 37.2, *p* < 0.00001. *p < 0.05, significant responses from Pre. ^†^ *p* < 0.05, L-CYSee versus vehicle or L-cysteine.

**FIGURE 3:**
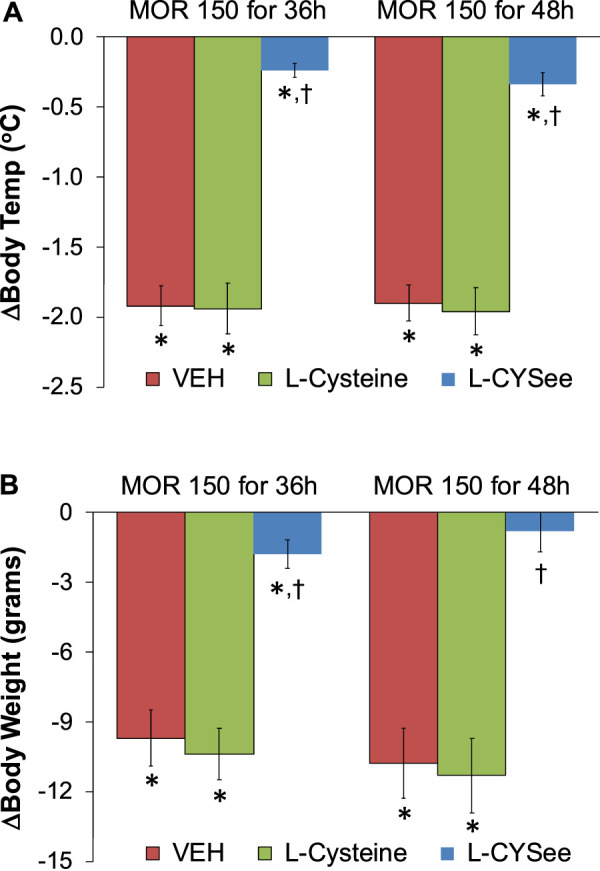
Arithmetic changes in body temperatures **(A)** and body weights **(B)** elicited by a bolus injection of NLX (1.5 mg/kg, IP) in rats treated for 36 h with a subcutaneous depot of morphine (150 mg/kg) or for 48 h with a subcutaneous depot of morphine (150 mg/kg) with continuous infusion of vehicle (saline, 20 μL/h, IV) or L-cysteine (20.8 μmol/kg/h, IV) or L-CYSee (20.8 μmol/kg/h, IV) for 36 h or beginning after 36 h for 12 h. The data are presented as mean ± SEM. There were nine rats in each group. Between-group ANOVA statistics: body temperature: 36 h: F_2,24_ = 51.4, *p* < 0.00001; 48 h: F_2,24_ = 50.1, *p* < 0.00001; body weight 36 h: F_2,24_ = 22.8, <0.00001; 48 h: F_2,24_ = 18.6, *p* < 0.00001. *p < 0.05, significant responses from Pre. ^†^ *p* < 0.05, L-CYSee versus vehicle or L-cysteine.

**FIGURE 4:**
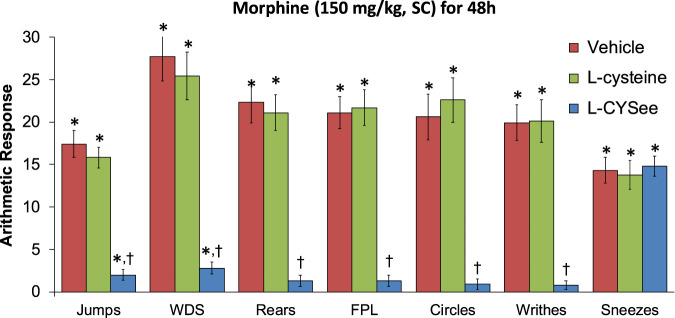
Withdrawal behaviors elicited by a bolus injection of NLX (1.5 mg/kg, IP) in rats treated for 48 h with a subcutaneous depot of morphine (150 mg/kg) along with continuous infusion of vehicle (saline, 20 μL/h, IV) or L-cysteine (20.8 μmol/kg/h, IV) or L-cysteine ethylester (L-CYSee, 20.8 μmol/kg/h, IV) that began after 36 h of morphine administration. Withdrawal signs: jumps—all four paws off the floor; WDS-wet-dog shakes; rears—rearing on hind legs; FPL—episodes of fore-paw licking; circles-a 360° rotation; writhes—full-body contortion; sneezes—abrupt expulsion of air. The data are presented as mean ± SEM. There were nine rats in each group. Between-group ANOVA statistics: jumps: F_2,24_ = 48.8, *p* < 0.00001; WDS: F_2,24_ = 33.9, *p* < 0.00001; rears: F_2,24_ = 38.7, <0.00001; FPL: F_2,24_ = 42.3, *p* < 0.00001; circles: F_2,24_ = 29.4, *p* < 0.00001; writhes: F_2,24_ = 34.0, *p* < 0.00005; sneezes: F_2,24_ = 0.11, *p* = 0.89. *p < 0.05, significant responses from Pre. ^†^ *p* < 0.05, L-CYSee versus vehicle or L-cysteine.

**FIGURE 5:**
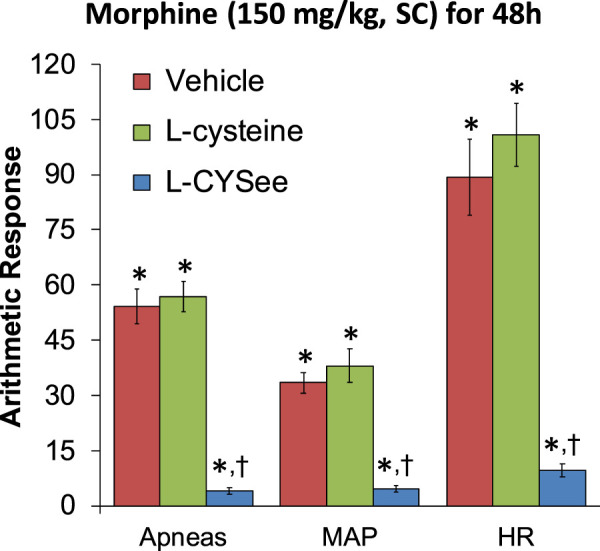
Incidence of apneas (>1.5 s between breaths) and increases in mean arterial blood pressure (MAP, mmHg) and heart rate (HR, beats/min) elicited by a bolus injection of NLX (1.5 mg/kg, IV) in rats treated for 48 h with a subcutaneous depot of morphine (150 mg/kg) along with the continuous infusion of vehicle (saline, 20 μL/h, IV), L-cysteine (20.8 μmol/kg/h, IV), or L-cysteine ethyl ester (L-CYSee, 20.8 μmol/kg/h, IV) that began at 36 h of morphine administration for 12 h. The data are presented as mean ± SEM. There were nine rats in each group. Between-group ANOVA statistics: MAP: F_2,24_ = 33.3, *p* < 0.00001; HR: F_2,24_ = 41.8, *p* < 0.00001; apneas: F_2,24_ = 69.9, *p* < 0.00001. *p < 0.05, significant responses from Pre. ^†^ *p* < 0.05, L-CYSee versus vehicle or L-cysteine.

The directly recorded and arithmetically derived parameters were statistically analyzed. All data are presented as mean ± SEM and were evaluated using one-way ANOVA, followed by Bonferroni corrections for multiple comparisons between means using the error mean square terms from each ANOVA analysis (Wallenstein et al., 1980; Ludbrook, 1998; McHugh, 2011), as detailed previously (Getsy et al., 2023a; Getsy et al., 2023b). A *p* < 0.05 value denoted the initial level of statistical significance that was modified according to the number of comparisons between means, as described by Wallenstein et al. (1980). The modified *t-*statistic is t = (mean group 1—mean group 2)/[s x (1/n_1_ + 1/n_2_)^1/2^], where s^2^ = the mean square within groups obtained from ANOVA (the square root of this value is used in the modified *t*-statistic formula), and n_1_ and n_2_ are the number of rats in each group under comparison. Based on Bonferroni’s inequality, a conservative critical value for modified *t*-statistics can be obtained from the tables of *t*-distribution using a significance level of P/m, where m is the number of comparisons to be made between groups (Winer, 1971). The degrees of freedom are those of the mean square for within group variation from the ANOVA table. The critical Bonferroni value can be approximated from the tables of the normal curve by t* = z + (z + z^3^)/4n, with n being the degrees of freedom and z being the critical normal curve value for P/m (Wallenstein et al., 1980; Ludbrook, 1998; McHugh, 2011). Wallenstein et al. (1980) first demonstrated that the Bonferroni procedure is preferable for general use because it provides critical values that are lower than those of other procedures when the number of comparisons can be limited and will be slightly larger than those of other procedures if many comparisons are made. Statistical analyses were performed with the aid of GraphPad Prism software (*GraphPad Software*, Inc., La Jolla, CA). F- and *P*-statistics associated with the ANOVA analyses of the data in Figures 1–5 are given in the respective figure legends.

## Results

### L-CYSee infusion prevents physical dependence on morphine—36-h studies

The behavioral withdrawal phenomena elicited by the injection of NLX (1.5 mg/kg, IP) in rats that were receiving morphine (150 mg/kg, SC), plus a continuous infusion of the vehicle (saline, 20 μL/h, IV) or L-cysteine (20.8 μmol/kg/h, IV) or L-CYSee (20.8 μmol/kg/h, IV) for 36 h are shown in Figure 1. As can be seen, the injection of NLX to rats receiving the infusion of the vehicle elicited substantial increases in jumping behavior (jumps), wet-dog shakes (WDS), rearing behavior (rears), fore-paw licking (FPL), circling behavior (circles), full-body writhing (writhes), and sneezing (sneezes). These responses were similar in magnitude in rats that were receiving an infusion of L-cysteine. In contrast, NLX-precipitated phenomena (except for sneezing) were dramatically diminished in rats that were receiving the continuous infusion of L-CYSee. In addition, the NLX-precipitated withdrawal signs were fully expressed in rats that were receiving an infusion of L-SERee (Table 1). Note that we did not pursue L-SERee studies to see whether it affected the expression of apneic events, and elevations in MAP and heart rate elicited by the injection of NLX.

**TABLE 1:** Naloxone-precipitated withdrawal signs in morphine-treated rats receiving continuous infusion of vehicle or L-serine ethyl ester for 36 h (prevention) or continuous infusion of vehicle or L-serine ethyl ester that began after 36 h of morphine administration and lasted for 12 h (reversal).

	Prevention of dependence 36 h morphine		Reversal of dependence 48 h morphine	
	Vehicle	L-SERee	Vehicle	L-SERee
Number of rats	9	9	9	9
Body weights (g)	335 ± 2	334 ± 1	335 ± 1	334 ± 2
Jumps	11.8 ± 1.7	12.1 ± 1.4	17.4 ± 1.6	20.1 ± 2.1
Full-body wet-dog shakes	17.0 ± 2.0	21.7 ± 2.7	27.7 ± 2.9	34.3 ± 3.2
Rearing behaviors	16.8 ± 2.5	18.0 ± 1.8	22.3 ± 2.4	25.4 ± 1.7
Fore-paw licking	14.2 ± 2.1	16.4 ± 1.8	21.1 ± 1.9	26.2 ± 2.6
360° circling behavior	14.3 ± 1.9	17.4 ± 2.1	20.6 ± 2.7	23.1 ± 2.3
Full-body writhes	13.9 ± 2.2	18.3 ± 2.4	19.9 ± 2.1	23.4 ± 2.3
Sneezes	8.9 ± 1.4	10.4 ± 1.5	14.3 ± 1.5	18.3 ± 2.1

L-SERee, L-serine ethyl ester. The data are presented as mean ± SEM. There were no between-group differences in body weights or withdrawal signs (*p* > 0.05, for all comparisons).

The increases in apneic events and elevations in MAP and heart rate, elicited by the injection of NLX (1.5 mg/kg) in rats that were receiving morphine (150 mg/kg, SC) and continuous infusion of vehicle or L-cysteine or L-CYSee are summarized in Figure 2. The injection of NLX in rats that were receiving the vehicle elicited substantial increases in the number of apneas and increases in MAP (sustained hypertension) and in heart rate (HR, tachycardia). These NLX-precipitated events were similar in magnitude in the rats that were receiving the infusion of L-cysteine. In contrast, the NLX-precipitated withdrawal phenomena were markedly reduced in rats receiving continuous infusion of L-CYSee. Actual values for MAP and heart rate before and after injection of NLX in morphine-treated rats receiving infusions of vehicle, L-cysteine, or L-CYSee are shown in Supplementary Table S1. Resting MAP and heart rate values before the injection of NLX were similar in the three groups of rats.

The changes in body temperatures and body weights elicited by the injection of NLX (1.5 mg/kg) in rats that were receiving morphine (150 mg/kg, SC) for 36 h, and continuous infusion of vehicle or L-cysteine or L-CYSee are summarized in Figure 3. The injection of NLX elicited marked decreases in body temperatures and body weights that were similar in magnitude in rats receiving infusion of vehicle or L-cysteine. The NLX-induced responses were markedly smaller in the rats that were receiving an infusion of L-CYSee. Actual body temperatures and body weights before and after injection of NLX in rats receiving morphine for 36 hours with co-infusions of vehicle, L-cysteine, or L-CYSee are shown in Supplementary Table S2. Resting body temperature and body weight values before the injection of NLX were similar in the three groups of rats. After 36 h of morphine treatment, body temperatures were elevated by just over 0.5°C in rats receiving infusions of vehicle or L-cysteine. Body temperature was not increased in rats receiving L-CYSee. The injection of NLX elicited substantial decreases in body temperatures and body weights in the vehicle- or L-cysteine-infusion groups, and much smaller responses in the rats receiving the infusion of L-CYSee.

### L-CYSee reversal of physical dependence on morphine—48-h studies

The behavioral withdrawal phenomena elicited by the injection of NLX (1.5 mg/kg, IP) in rats that were receiving morphine (150 mg/kg, SC) for 48 h plus a continuous infusion of the vehicle (saline, 20 μL/h, IV) or L-cysteine (20.8 μmol/kg/h, IV) or L-CYSee (20.8 μmol/kg/h, IV) beginning after 36 h are shown in Figure 4. The injection of NLX into rats receiving the vehicle infusion elicited substantial increases in withdrawal behaviors. These responses were similar in magnitude in rats receiving infusion of L-cysteine, whereas withdrawal phenomena (except for sneezing) were dramatically diminished in rats receiving infusion of L-CYSee for 12 h. NLX-precipitated withdrawal signs in rats receiving an infusion of L-SERee were similar to those receiving infusion of vehicle (Table 1).

The increases in apneic events and elevations in MAP and heart rate, elicited by the injection of NLX (1.5 mg/kg, IP) in rats receiving morphine (150 mg/kg, SC) for 48 h and continuous infusion of vehicle or L-cysteine or L-CYSee beginning at 36 h are shown in Figure 5. NLX elicited substantial increases in apneas and elevations in MAP (hypertension) and in heart rate (HR, tachycardia) in rats receiving infusion of vehicle, and similar responses in rats receiving infusion of L-cysteine. In contrast, NLX-precipitated withdrawal phenomena were markedly reduced in rats receiving L-CYSee infusion. Actual values for MAP and heart rate before and after the injection of NLX in morphine-treated rats receiving infusion of vehicle, L-cysteine, or L-CYSee are shown in Supplementary Table S3. Resting MAP and heart rate values before the injection of NLX were similar in the three groups.

The changes in body temperatures and body weights elicited by the injection of NLX (1.5 mg/kg) in rats receiving morphine (150 mg/kg, SC) and infusion of vehicle or L-cysteine or L-CYSee that began 36 h after morphine administration and lasted for 12 h are summarized in Figure 3. The injection of NLX elicited pronounced decreases in body temperatures and body weights that were similar in magnitude in rats receiving vehicle or L-cysteine. These withdrawal responses were markedly smaller in rats receiving L-CYSee. Full details of the body temperatures and body weight before and after injection of NLX in these morphine-treated rats that were receiving infusions of vehicle, L-cysteine, or L-CYSee are shown in Supplementary Table S4. Resting body temperature and body weight values before the injection of NLX were similar in the three groups of rats. After 48 h of morphine treatment, body temperatures were elevated by just over 0.5°C in rats receiving infusions of vehicle or L-cysteine. The body temperatures were not elevated in rats receiving the infusion of L-CYSee. Body weights were similar in all three groups. The injection of NLX elicited substantial decreases in body temperatures and body weights in the vehicle- or L-cysteine-infusion groups, and much smaller responses in the rats receiving infusion of L-CYSee.

## Discussion

The first set of major observations of this study was that co-infusion of the L-thiol ester, L-CYSee, markedly reduced the expression of multiple withdrawal signs (behavioral, cardiorespiratory, body weight loss, and hypothermia) elicited by the injection of the opioid receptor antagonist, NLX, in male Sprague Dawley rats treated for 36 h with slow-release morphine emulsion. The behavioral withdrawal signs indicative of the rats having become physically dependent on morphine, such as jumping, wet-dog shakes, rearing, fore-paw licking, circling, writhing, and sneezing (rapid expulsions of air), as well as decreases in body weight and body temperature, were consistent with previous reports published using this same slow-release morphine model (Lee and Fennessy, 1970; Laska and Fennessy, 1976; Laska and Fennessy, 1977; Laska and Fennessy, 1978; Lewis et al., 1988b), and with a wide variety of other administration protocols used to induce morphine dependence (Hutchinson et al., 2007; Lopez-Gimenez and Milligan, 2010; Morgan and Christie, 2011; Nielsen and Kreek, 2012). The increases in MAP and heart rate elicited by NLX are new findings in our morphine-dependence model, but are in full agreement with evidence that NLX-precipitated withdrawal is associated with hypertension and tachycardia in experimental animals (Buccafusco, 1983; Buccafusco, 1990; Buccafusco et al., 1984; Marshall and Buccafusco, 1985; Dixon and Chang, 1988; Chang and Dixon, 1990; Delle et al., 1990; Baraban et al., 1993) and humans (Newlin et al., 1992; Purssell et al., 1995; Walsh et al., 2003; Levin et al., 2019; Balshaw et al., 2021; Isoardi et al., 2022; Lee et al., 2022). This is due to globalized activation of the sympathetic nervous system. Finally, our finding that NLX elicited a substantial increase in apneic events is new to our morphine-dependence model, but also consistent with findings in rats (Baraban et al., 1993; Baldo, 2022) and humans (Schwarzer et al., 2015; Zamani et al., 2020; Wilson et al., 2023). The inability of L-cysteine to modify the NLX-precipitated withdrawal phenomena certainly suggests that the efficacy of L-CYSee involves the entry of this cell-penetrant L-thiol ester (Goto et al., 1983; Hisadome et al., 1986a; Hisadome et al., 1986b; Hisadome et al., 1988; Servin et al., 1988; Hisadome et al., 1990; Schöneich et al., 1992; Hobbs et al., 1993; Fukui et al., 1994; Ding and Demple, 1998; Galanakis et al., 2004; Mosier-Boss and Lieberman, 2005; Perissinotti et al., 2005; Defonsi Lestard et al., 2013; Mendoza et al., 2013; Arias et al., 2019; Lewis et al., 2022) into neurons involved in the acquisition of physical dependence/addiction on morphine (Laschka et al., 1976a; Laschka et al., 1976b; Laschka and Herz, 1977; Koob, 1987; Saiepour et al., 2001; Glass, 2010; Gardner, 2011; Glass, 2011). Moreover, the inability of L-SERee to prevent the acquisition of physical dependence on morphine indicates that the sulfur atom is vital to the ability of L-CYSee to prevent the intracellular processes within the brain by which morphine induces physical dependence (Deslandes et al., 2002; Gardner, 2011; Koob and Volkow, 2016; Volkow et al., 2019; Koob, 2020; Sakloth et al., 2020).

At present, we do not know how L-CYSee prevents the development of physical dependence on morphine. The mechanisms by which L-thiol esters exert their biological effects are likely to be multi-factorial and possibly include 1) direct binding of L-CYSee to plasma membrane/intracellular proteins, such as ion channels, receptors, and enzymes that alter the activities of the proteins by mechanisms not associated with the changes in the redox status of the proteins; 2) formation of thiol adducts, such as D-glucose-L-cysteine (Wróbel et al., 1997; Szwergold, 2006; Li et al., 2015) and mixed disulfides (Wilcken and Gupta, 1979; Lash and Jones, 1985; Turell, et al., 2013) in the blood; 3) modulation of redox status (e.g., reduction in L-cystine to L-cysteine), and the activity of plasma membrane proteins, such as Kv_1.2_ K^+^-channels (Baronas et al., 2017), and after entry into cells, redox modulation of functional intracellular proteins (Bogeski et al., 2011; Bogeski and Niemeyer, 2014; O-Uchi et al., 2014; Gamper and Ooi, 2015; Gao et al., 2017; Garcia et al., 2018); 4) formation of S-thiolated proteins, such as S-cysteinylated, S-cysteinylglycinylated, and S-glutathionylated proteins, in plasma membranes of cells (Winkler et al., 2007; Rossi et al., 2009; Auclair et al., 2013; Belcastro et al., 2017; Ghezzi and Chan, 2017; Bonifácio et al., 2021); 5) conversion of L-CYSee to L-cysteine by membrane-associated esterases (Butterworth et al., 1993; Nishida et al., 1996), which then enters into multiple metabolic pathways including those that generate hydrogen sulfide via the sequential actions of L-cysteine aminotransferase and cystathionine γ-lyase in peripheral and central tissues (Kimura, 2014; Kimura, 2017; Bełtowski, 2019), including the carotid bodies (Prabhakar, 2012); 6) conversion of L-thiol esters to cysteine sulfenic, sulfinic, and sulfonic via cysteine dioxygenase (Yamaguchi and Hosokawa, 1987; Joseph and Maroney, 2007; Stipanuk et al., 2009; Stipanuk et al., 2011); and 7) formation of S-nitroso-L-cysteine, an endogenous S-nitrosothiol (Myers et al., 1990; Bates et al., 1991; Seckler et al., 2020), with many substantial roles in intracellular signaling cascades (Lipton et al., 1993; Foster et al., 2009; Seth and Stamler, 2011; Stomberski et al., 2019; Gaston et al., 2020), including those controlling the cardiorespiratory function (Davisson et al., 1996; Davisson et al., 1997; Ohta et al., 1997; Lipton et al., 2001; Gaston et al., 2006; Lewis et al., 2006; Gaston et al., 2020) and those involved in the attenuation of opioid induced respiratory depression (OIRD) (Getsy et al., 2022a; Getsy et al., 2022b). Any or all of these mechanisms (and possibly those not mentioned) may interact with signaling pathways involved in the acquisition of physical dependence on opioids, such as morphine, and the expression of the NLX-precipitated withdrawal syndrome, including those involving N-methyl D-aspartate (NMDA) glutamatergic receptors (Buccafusco et al., 1995; Herman et al., 1995; Rasmussen, 1995; Noda and Nabeshima, 2004; Glass, 2011; Fluyau et al., 2020), muscarinic receptors (Marshall and Buccafusco, 1987; Holland et al., 1993), corticotropin-releasing factor (CRF) receptor (CRF1) (García-Carmona et al., 2015), tachykinin receptors (Michaud and Couture, 2003), voltage-gated Ca^2+^-channels (Tokuyama et al., 1995; Dogrul et al., 2002; Esmaeili-Mahani et al., 2008; Alboghobeish et al., 2019), adenylyl cyclase superactivation and opioid receptor phosphorylation (Avido-Reiss et al., 1996; Avido-Reiss et al., 1997; Wang et al., 1999; Eckhardt et al., 2000), oxidative stress (Xu et al., 2006; Mori et al., 2007; Abdel-Zaher et al., 2013; Mansouri et al., 2020; Ward et al., 2020; Houshmand et al., 2021), and the nitric oxide-cGMP signaling cascade (Adams et al., 1993; Cappendijk et al., 1993; Majeed et al., 1994; Vaupel et al., 1995a; Buccafusco et al., 1995; Vaupel et al., 1995b; Herman et al., 1995; Leza et al., 1995; 1996; London et al., 1995; Dambisya and Lee, 1996; Bhatt and Kumar, 2015; Tsakova et al., 2015; Sackner et al., 2019; Gledhill and Babey, 2021). Since L-CYSee blunted the expression of all NLX-precipitated behavioral (except for sneezing), physical (body weight loss and hypothermia), and cardiorespiratory (hypertension, tachycardia, and incidence of apneas) phenomena, it is tempting to assume that L-CYSee interrupts fundamental intracellular processes that are essential for the development of physical dependence on morphine.

The second set of novel findings was that the introduction of L-CYSee infusion 36 h into the morphine administration period appeared to reverse the established physical dependence on the opioid, as assessed at 48 h (i.e., within 12 h of giving continuous infusion of L-CYSee). Specifically, NLX-precipitated behavioral phenomena (except for sneezing), hypertension, tachycardia, apneas, hypothermia, and body weight loss were markedly fewer in the rats that had received L-CYSee for 12 h. Again, the lack of effect of L-cysteine and L-SERee suggests that the intracellular delivery of L-CYSee and its sulfur atom (and associated thiol chemistry) is essential to the ability of the L-thiol ester to reverse the established physical dependence on morphine. Again, the mechanism of action for how L-CYSee reverses physical dependence on morphine is not known, but any of the mechanisms discussed in the previous paragraph, including its potent antioxidant properties, may be involved. The well-known therapeutics and bioactive compounds that reverse established physical dependence include: L-histidine and certain histamine receptor sub-type agonists (Wong and Roberts, 1976), such as melatonin (Raghavendra and Kulkarni SK, 1999; Raghavendra and Kulkarni SK, 2000); the antioxidant, quercetin (Singh et al., 2002; Naidu et al., 2003); the serotonin-reuptake inhibitor, fluoxetine (Singh et al., 2003); the nitric oxide synthase inhibitor, L-N^G^-nitroarginine methyl ester (Naidu et al., 2003; Sing et al., 2003); inhibitors of Ca^2+^/calmodulin-dependent protein kinase II (Wang et al., 2003; Tang et al., 2006); the β_2_-AR antagonist, butoxamine (Liang et al., 2007); adrenomedullin receptor antagonists (Wang et al., 2011); the antipsychotic (dopamine D2 receptor antagonist), haloperidol (Yang et al., 2011); and positive allosteric modulators of AMPA (α-amino-3-hydroxy-5-methyl-4-isoxazolepropionic acid) glutamatergic receptors (Hu et al., 2018). The mechanism(s) of action for how L-CYSee reverses established physical dependence on morphine is of great clinical relevance, and opens the way for future studies on this and other bioactive L,D-thiol esters and related compounds (Baby et al., 2021; Baby et al., 2021; Gaston et al., 2021; Jenkins et al., 2021; Getsy et al., 2022a; Getsy et al., 2022b; Getsy et al., 2022c; Getsy et al., 2022d; Getsy et al., 2022e; Getsy et al., 2022f; Lewis et al., 2022) with respect to their ability to reverse physical dependence on morphine and other opioids, including heroin and fentanyl.

Many questions remain regarding the potential use of L-thiol esters as therapeutics for key clinical problems associated with opioid analgesia in humans, including: 1) if L-CYSee attenuates/blocks self-administration of opioids in OUD patients, adding it to prescription opioids may result in lower drug abuse or addiction potential; 2) if L-CYSee attenuates or blocks development of physical dependence on opioids, then adding it to prescription opioids will minimize and may potentially eliminate physical dependence in individuals who receive opioids for a long-term basis, such as everyday, all day, for weeks/months; 3) if L-CYSee attenuates/blocks tachyphylaxis to opioid analgesia or hyperalgesia caused by opioids, then the addition of L-CYSee to prescription opioids will maintain their analgesic efficacy over long periods of time, eliminating the development of tolerance, need for escalating doses, and potential complications of hyperalgesia; 4) if L-CYSee has several of the advantageous effects observed in rodents, then adding it to opioid analgesics would multiply the beneficial aspects of the opioids; 5) if L-CYSee prevents the development of physical dependence, and specifically, if it is introduced to an individual with physical dependence and attenuates/blocks opioid withdrawal, it could be used as an outpatient/inpatient medication to manage opioid withdrawal in those who are iatrogenically physically dependent (long-term opioid prescriptions) or those who are addicted and physically dependent; 6) if L-CYSee attenuates/blocks euphoria and/or the development of physiological dependence to opioids, then it would be a good medication for medication-assisted treatment (MAT) and a potentially good drug for harm reduction interventions in people with OUD who are not interested in the psychosocial aspects of counseling and treatment; 7) as some patients with a history of OUD who are currently sober need opioids for the treatment of acute or chronic pain syndromes, this L-thiol ester, if it attenuates or blocks euphoria and physical dependence, could be added to opioid analgesics when given to people with a history of OUD, thereby eliminating the risk of opioid analgesic-precipitating euphoria, drug cravings, and their markedly increased risk of relapse; 8) if L-CYSee attenuates/blocks euphoria from chemically mediated dopamine surges within the ventral tegmentum, nucleus accumbens, or medial prefrontal cortex, where brain rewarding euphoria-producing dopamine surge happens from all drugs of abuse/addiction (Deslandes et al., 2002; Gardner, 2011; Koob and Volkow, 2016; Volkow et al., 2019; Koob, 2020; Sakloth et al., 2020), then it will be useful in the treatment of OUD and other SUDs; and 9) if L-CYSee attenuates/blocks euphoria from chemically mediated dopamine surges, it could be combined with or added to all controlled prescription drugs, resulting in an abuse-resistant or non-abusable form of prescribed opioids, benzodiazepines, and psychostimulants. In relation to point (1), we recently showed that co-administration of the D-isomer, D-cysteine ethyl ester, with fentanyl prevents the development of fentanyl-induced conditioned place preference in both male and female rats (Knauss et al., 2023). Thus, L,D-thiol esters likely reduce the rewarding properties of opioids and reduce their addictive potential.

A final caveat to translating these findings to humans lies in the genetic variation of humans (e.g., 5–6 million SNPs between any two individuals) in comparison to the total variation found in the outbred Sprague Dawley rats tested here (Gileta et al., 2022). This lack of genetic variation in preclinical models is one explanation for the inability of many findings to translate across species (Garner, 2014; Zuberi and Lutz, 2016). The pharmacogenetics of opioids have been studied, and numerous pharmacokinetic molecules, such as COMT, OPRM1, CYP2D6, and ABCB1, have been identified that are useful in clinical application (Owusu Obeng et al., 2017). In animal models, opioid withdrawal specifically has been studied using the C57BL/6J x DBA2 recombinant inbred lines, where a locus on chromosome 16 was identified as affecting the locomotor behavior of naloxone-precipitated withdrawal (Philip et al., 2010). In another population, 129 × C57BL/6 cross-identified loci on chromosomes 1, 5, and 10 were involved in withdrawal jumping frequencies (Kest et al., 2004). Testing the efficacy of drugs using a population of outbred mice containing approximately 45 million segregating SNPs, such as the *diversity outbred* mice (Saul et al., 2019), will increase the likelihood that the drug will translate within species before traversing across species.

## Study limitations

A limitation of the present study is that we have not examined the efficacy of lower doses of L-CYSee to prevent or reverse morphine-induced physical dependence. Finding out the lower limit is key to minimizing potential adverse biological effects that were not monitored in the present study. In particular, we have not determined whether the co-administration of L-CYSee alters the analgesic actions of morphine, although we have reported that L-CYSee (Lewis et al., 2022), L-cysteine methyl ester (Getsy et al., 2022a), and other thiolesters and related compounds (Baby et al., 2021; Baby et al., 2021; Gaston et al., 2021; Jenkins et al., 2021; Getsy et al., 2022b; Getsy et al., 2022c; Getsy et al., 2022d; Getsy et al., 2022e; Getsy et al., 2022f) prevent and/or reverse the actions of morphine and fentanyl on ventilatory parameters, arterial blood–gas chemistry, and alveolar-arterial gradient in freely-moving rats without compromising opioid-induced analgesia or sedation. Synthetic opioids, especially fentanyl, are playing a major and ever-increasing role in the current opioid crisis (Arendt, 2021; Deo et al., (2021), and future studies must determine whether L-CYSee can overcome and reverse physical dependence on fentanyl. A concern about the study protocol regarding the ability of L-CYSee to reverse established morphine dependence, was the short time (12 h) between the surgery to initiate the intravenous infusions (implantation of minipumps under isoflurane anesthesia), and the administration of NLX to precipitate withdrawal. Despite the robust nature of the NLX-induced responses in rats that received intravenous infusions of vehicle, L-cysteine, or L-SERee for 12 h, it is possible that the lingering effects of anesthesia affected the expression of the withdrawal phenomena. Another important limitation of our study is the lack of data about the efficacy of L-CYSee in preventing/reversing physical dependence in female rats. This is especially important because 1) opioids exert qualitatively and quantitatively different pharmacological (e.g., ventilation, analgesia) responses in females compared to males (Dahan et al., 1998; Sarton et al., 1998; Bodnar and Kest, 2010); 2) there are many sex-specific differences in opioid receptor signaling (Bryant et al., 2006; Hosseini et al., 2011); 3) pronounced sex differences in development of opioid hyperalgesia, tolerance and withdrawal (Bodnar and Kest, 2010); and 4) several major behavioral sex differences in the expression and treatment of OUDs (Huhn et al., 2019; Davis et al., 2021; Knouse and Briand, 2021). The lack of understanding about the molecular mechanisms by which L-CYSee affects the acquisition/reversal of morphine dependence is a limitation that needs to be addressed. In addition to potential direct interactions with yet to be defined functional proteins, potential mechanisms of action of L-CYSee may involve 1) direct binding to putative L,D-cysteine-binding protein, such as myristoylated alanine-rich C-kinase substrate (Semenza et al., 2021), 2) interruption of μ-OR-β-arrestin-coupled cell signaling processes to spare the antinociceptive G-protein-dependent actions of morphine (Schmid et al., 2017; Grim et al., 2020), and/or 3) potential conversion of L-CYSee to S-nitroso-L-CYSee or S-nitroso-L-cysteine by S-nitrosylation of the sulfur atom in L-thiol esters via processes requiring nitric oxide synthase (Perissinotti et al., 2005; Hess and Stamler, 2012; Stomberski et al., 2019; Seckler et al., 2020; Seckler et al., 2022), which may act via an intracellular penetrating mechanism(s) (Clancy et al., 2001). To test the possibility that L-CYSee elicits the production of S-nitrosylated versions of the L-thiol ester, we are determining whether intravenous injections of L-CYSee increase the production of S-nitrosylated species in the blood, peripheral tissues, and brain via the use of an ultra-sensitive capacitive sensor (Seckler et al., 2017), and whether such injections of L-CYSee increase the expression of NADPH diaphorase in the brain and peripheral structures, on the basis that NADPH diaphorase is used to visualize free S-nitrosothiols and S-nitrosylated proteins in aldehyde-treated tissue (Seckler et al., 2020). S-nitrosothiols, such as S-nitroso-L-cysteine and S-nitroso-L-glutathione, play important roles in ventilatory control processes in the brainstem, circulating red blood cells, and peripheral structures, such as the carotid bodies (Lipton et al., 2001; Gaston et al., 2006; Palmer et al., 2013; Gaston et al., 2014; Palmer et al., 2015; Gaston et al., 2020). The possibility that the conversion of L-CYSee to S-nitroso-L-CYSee is responsible for the effects observed in this study would add to our understanding of the pharmacology of L–S-nitrosothiols (Davisson et al., 1996; Lewis et al., 1996; Travis et al., 1996; Davisson et al., 1997; Travis et al., 1997; Lewis et al., 2005; Gaston et al., 2006; Lewis et al., 2006; Gaston et al., 2020). Another limitation that certainly needs addressing is our lack of information about the blood and tissue distribution resulting from the infusion of L-CYSee in the presence or absence of morphine, although L-CYSee can be readily detected in plasma, and peripheral and central tissues upon acute administration to naïve rats (Servin et al., 1998). We intend to perform pharmacokinetics analyses of L-CYSee distribution in brain regions involved in the acquisition of opioid dependence, such as the medial prefrontal cortex (Deslandes et al., 2002; Gardner, 2011; Koob and Volkow, 2016; Volkow et al., 2019; Koob, 2020; Sakloth et al., 2020), using our liquid chromatography–mass spectrometry methodology (Altawallbeh et al., 2019).

## Conclusion

This study demonstrates that systemic infusion of the membrane-permeable L-thiol ester, L-CYSee, prevents the development of physical dependence on morphine in male Sprague Dawley rats by mechanisms dependent on thiol biochemistry. In addition, this study demonstrates that L-CYSee reverses established dependence on morphine in rats also by thiol-dependent processes. Delineating the exact thiol-dependent signaling pathways will add greatly to our understanding of the processes by which opioids induce dependence, and how the bioactive L-thiol esters exert their effects. Our study was spurred by the ground-breaking work of Trivedi, Deth, and others, which added greatly to our understanding of the mechanisms that opioids cause physical dependence and psychological addiction (Trivedi et al., 2014; Trivedi and Deth, 2015; Trivedi et al., 2015). Their evidence that morphine may cause dependence/addiction by blocking the entry of L-cysteine into neurons inhibiting the EAA3/EAAC1 transporters (Trivedi et al., 2014) prompted our studies with the membrane-permeable, L-thiol ester, L-CYSee. The findings that L-CYSee markedly reduced the majority of NLX-precipitated withdrawal phenomena suggests that decreased levels of L-cysteine entry into cells plays a key role in establishing physical dependence on morphine. The lone withdrawal phenomenon that was not ameliorated by L-CYSee was sneezing, a key feature of the opioid withdrawal response in humans (Ostrea et al., 1975; Specker et al., 1998; Gaalema et al., 2012; Lofwall et al., 2013) and experimental animals (Hendrie, 1985; Liu et al., 2007; Singh et al., 2015). We are currently trying to understand the current state of knowledge about the neural mechanisms responsible for sneezing (Batsel and Lines, 1975; Undem et al., 2000; Li et al., 2021; Ramirez et al., 2022) to see if that can give insights into the signaling pathways that are/are not involved in the actions of L-CYSee. The present findings add to our knowledge about the efficacy of L,D-thiol esters, such as L-CYSee (Lewis et al., 2022) L-GSHee (Jenkins et al., 2021), D-CYSee (Getsy et al., 2022c; Getsy et al., 2022d), D-cystine di(m)ethyl ester (Gaston et al., 2021), and the free radical-superoxide anion scavenger, Tempol (Baby et al., 2021a; Baby et al., 2021b), on the pharmacological actions of opioids.

## Data Availability

The raw data supporting the conclusion of this article will be made available by the authors, without undue reservation.

## Abbreviations

AMPA, α-amino-3-hydroxy-5-methyl-4-isoxazolepropionic acid; CRF, corticotropin-releasing factor; EAA3, excitatory amino acid transporter; IP, intraperitoneal; IV, intravenous; L-CYSee, L-cysteine ethyl ester; L-NAC, N-acetyl-L-cysteine; Kv_1.2_ K^+^-channels, voltage-gated K^+^_1.2_ channels; L-SERee, L-serine ethyl ester; μ-OR, μ-opioid receptor; MAP, mean arterial blood pressure; NMDA, N-methyl D-aspartate; NLX, naloxone hydrochloride; OIRD, opioid-induced respiratory depression; OUD, opioid use disorder; SAM, S-adenosyl-methionine; SAH, S-adenosyl-homocysteine; SC, subcutaneous; SUD, substance use disorder.
