# Supplementary material for: L-cysteine ethyl ester prevents and reverses acquired physical dependence on morphine in male Sprague Dawley rats
Source: Front Pharmacol. 2023 Dec 4;14:1303207. doi: 10.3389/fphar.2023.1303207 (PMC10726967; doi:10.3389/fphar.2023.1303207)
Supplement: Supplementary file 1 [file Table1.docx]

**Supplementary File**

**L-cysteine ethyl ester prevents and reverses acquired physical dependence to morphine in male Sprague Dawley rats**

James N. Bates,^1,†^ Paulina M. Getsy,^2^ Gregory A. Coffee,^2^ Santhosh M. Baby,^3,‡^

Peter M. MacFarlane,^2^ Yee-Hsee Hsieh,^4^ Zackery T. Knauss,^5^ Jason A. Bubier,^6^ Devin Mueller^5^ and Stephen J. Lewis^2,7,8,^*

*^1^Department of Anesthesiology, University of Iowa Hospitals and Clinics, Iowa City, Iowa, USA*

*^2^Department of Pediatrics, Case Western Reserve University, Cleveland, Ohio, USA*

*^3^Section of Biology, Galleon Pharmaceuticals, Inc, Horsham, Pennsylvania, USA*

*^4^Division of Pulmonary, Critical Care and Sleep Medicine, Case Western Reserve University,*

*Cleveland, Ohio, USA*

*^5^Department of Biological Sciences, Kent State University, Kent, Ohio, USA*

*^6^Jackson Laboratories, Bar Harbor, Maine, USA*

*^7^Department of Pharmacology,* *Case Western Reserve University, Cleveland, Ohio, USA*

*^8^Functional Electrical Stimulation Center, Case Western Reserve University, Cleveland, Ohio, USA*

**^†^Present Address:** James N. Bates, Chief Medical Officer, *Atelerix Life Sciences Inc*. Address: 300 East Main Street, Suite 202 Charlottesville, Virginia 22902. Email: jbates@atelerixlifesciences.com. https://atelerixlifesciences.com

**^‡^Present address:** Translational Sciences Treatment Discovery, Galvani Bioelectronics, Inc, 1250 S Collegeville Rd, Collegeville, PA 1r9426, USA. Email: babysanthosh@gmail.com

***Corresponding Author:** Stephen J. Lewis, PhD. Department of Pediatrics, Division of Pulmonology, Allergy and Immunology, School of Medicine, Case Western Reserve University, 10900 Euclid Avenue, Cleveland, OH 44106-4984. Email: sjl78@case.edu

**Supplementary Table S1.** Changes in mean arterial blood pressure (MAP) and heart rate elicited by injection of naloxone HCl (NLX) in freely-moving male rats receiving morphine with co-infusion of vehicle or L-cysteine or L-CYSee for 36 hours.

| **Mean arterial blood pressure (mmHg)** | | | | |  | **Actual Values** | | | | |
| --- | --- | --- | --- | --- | --- | --- | --- | --- | --- | --- |
| **Emulsion** |  | **Infusion** |  | **Injection** |  | **Pre** |  | **+36h** |  | **Post-NLX** |
| Morphine |  | Vehicle |  | NLX |  | 112 ± 2 |  | 145 ± 3 |  | +33.3 ± 2.8* |
| Morphine |  | L-Cysteine |  | NLX |  | 114 ± 2 |  | 150 ± 4 |  | +36.2 ± 4.5* |
| Morphine |  | L-CYSee |  | NLX |  | 112 ± 2 |  | 117 ± 2 |  | +4.9 ± 1.1*^,†^ |
| **Heart rate (beats/min)** | | |  |  |  | **Actual Values** | | | | |
| **Emulsion** |  | **Infusion** |  | **Injection** |  | **Pre** |  | **+36h** |  | **Post-NLX** |
| Morphine |  | Vehicle |  | NLX |  | 354 ± 6 |  | 439 ± 7 |  | +84.8 ± 8.0* |
| Morphine |  | L-Cysteine |  | NLX |  | 358± 6 |  | 454 ± 8 |  | +96.0 ± 8.1* |
| Morphine |  | L-CYSee |  | NLX |  | 356 ± 6 |  | 368 ± 7 |  | +11.2 ± 2.3*^,†^ |

Responses elicited by the acute injection of NLX (1.5 mg/kg. IV) in rats treated for 36h with a subcutaneous depot of 150 mg/kg morphine and co-infusions of vehicle (20 μL/h, IV) or L-cysteine (20.8 μmol/kg/h, IV) or L-cysteine ethyl ester (L-CYSee, 20.8 μmol/kg/h, IV). There were 9 rats in each group. The data are presented as mean ± SEM. **p* < 0.05, significant responses from Pre. ^†^*p* < 0.05, L-CYSee *versus* vehicle or L-cysteine*.*

**Supplementary Table S2.** Changes in body temperatures and body weights elicited by the injection of naloxone HCl (NLX) in rats receiving morphine with co-infusion of vehicle, L-cysteine or L-CYSee for 36 hours.

| **Body Temperature (°C)** | | | |  |  | **Actual Values** | | | | |  | **ΔChange (°C)** | | |
| --- | --- | --- | --- | --- | --- | --- | --- | --- | --- | --- | --- | --- | --- | --- |
| **Emulsion** |  | **Infusion** |  | **Injection** |  | **Pre** |  | **+36h** |  | **Post-NLX** |  | **+36h vs Pre** |  | **NLX response** |
| Morphine |  | Vehicle |  | NLX |  | 37.5 ± 0.1 |  | 38.0 ± 0.1 |  | 36.1 ± 0.1 |  | +0.57 ± 0.08* |  | -1.92 ± 0.14* |
| Morphine |  | L-Cysteine |  | NLX |  | 37.6 ± 0.1 |  | 38.3 ± 0.1 |  | 36.3 ± 0.1 |  | +0.69 ± 0.11* |  | -1.94 ± 0.18* |
| Morphine |  | L-CYSee |  | NLX |  | 37.5 ± 0.1 |  | 37.6 ± 0.1 |  | 37.4 ± 0.1 |  | +0.09 ± 0.07^†^ |  | -0.24 ± 0.05*^,†^ |
| **Body Weight (grams)** | | |  |  |  | **Actual Values** | | | | |  | **ΔChange (grams)** | | |
| **Emulsion** |  | **Infusion** |  | **Injection** |  | **Pre** |  | **+36h** |  | **Post-NLX** |  | **+36h vs Pre** |  | **NLX response** |
| Morphine |  | Vehicle |  | NLX |  | 335 ± 2 |  | 336 ± 2 |  | 327 ± 1 |  | +0.9 ± 0.7 |  | -9.7 ± 1.2* |
| Morphine |  | L-Cysteine |  | NLX |  | 336 ± 1 |  | 338 ± 1 |  | 326 ± 2 |  | +0.4 ± 0.8 |  | -10.4 ± 1.1* |
| Morphine |  | L-CYSee |  | NLX |  | 336 ± 2 |  | 336 ± 2 |  | 334 ± 2 |  | +1.0 ± 0.6 |  | -1.8 ± 0.6*^,†^ |

Responses elicited by the acute injection of NLX (1.5 mg/kg, IP) in rats treated for 36h with a subcutaneous depot of 150 mg/kg morphine and co-infusions of vehicle (20 μL/h, IV) or L-cysteine (20.8 μmol/kg/h, IV) or L-cysteine ethyl ester (L-CYSee, 20.8 μmol/kg/h, IV) There were 9 rats in each group. The data are presented as mean ± SEM. **p* < 0.05, significant responses from Pre. ^†^*p* < 0.05, L-CYSee *versus* vehicle or L-cysteine*.*

**Supplementary Table S3.** Changes in mean arterial blood pressure and heart rate elicited by the injection of naloxone HCl (NLX) in rats receiving morphine for 48 hours with co-infusion of vehicle, L-cysteine or L-CYSee for 12 hours starting at 36 hours of morphine administration.

| **Mean arterial blood pressure (mmHg)** | | | | |  | **Actual Values** | | | | |
| --- | --- | --- | --- | --- | --- | --- | --- | --- | --- | --- |
| **Emulsion** |  | **Infusion** |  | **Injection** |  | **Pre** |  | **+48h** |  | **Post-NLX** |
| Morphine |  | Vehicle |  | NLX |  | 115 ± 2 |  | 148 ± 4 |  | +33.4 ± 2.8* |
| Morphine |  | L-cysteine |  | NLX |  | 113 ± 2 |  | 151 ± 3 |  | +38.0 ± 4.6* |
| Morphine |  | L-CYSee |  | NLX |  | 116 ± 2 |  | 121 ± 1 |  | +4.6 ± 1.0*^,†^ |
| **Heart rate (beats/min)** | | |  |  |  | **Actual Values** | | | | |
| **Emulsion** |  | **Infusion** |  | **Injection** |  | **Pre** |  | **+48h** |  | **Post-NLX** |
| Morphine |  | Vehicle |  | NLX |  | 361 ± 7 |  | 450 ± 8 |  | +89.3 ± 10.2* |
| Morphine |  | L-cysteine |  | NLX |  | 357 ± 7 |  | 458 ± 12 |  | +100.9 ± 8.6* |
| Morphine |  | L-CYSee |  | NLX |  | 362 ± 5 |  | 372 ± 6 |  | +9.6 ± 1.7*^,†^ |

Responses elicited by the acute injection of NLX (1.5 mg/kg, IV) in rats treated for 36h with a subcutaneous depot of 150 mg/kg morphine and co-infusions of vehicle (20 μL/h, IV) or L-cysteine (20.8 μmol/kg/h, IV) or L-cysteine ethyl ester (L-CYSee, 20.8 μmol/kg/h, IV) for 12 hours starting at 36 hours of morphine administration. There were 9 rats in each group. The data are presented as mean ± SEM. **p* < 0.05, significant responses from Pre. ^†^*p* < 0.05, L-CYSee *versus* vehicle or L-cysteine*.*

**Supplementary Table S4.** Changes in body temperatures and body weights elicited by the injection of naloxone HCl (NLX) in rats treated with morphine for 48 hours with co-infusion of L-CYSee or L-cysteine for 12 hours starting at 36 hours of morphine administration

| **Body Temperature (°C)** | | | |  |  | **Actual Values** | | | | |  | **ΔChange (°C)** | | |
| --- | --- | --- | --- | --- | --- | --- | --- | --- | --- | --- | --- | --- | --- | --- |
| **Emulsion** |  | **Infusion** |  | **Injection** |  | **Pre** |  | **+48h** |  | **Post-NLX** |  | **+48h vs Pre** |  | **NLX response** |
| Morphine |  | Vehicle |  | NLX |  | 37.5 ± 0.1 |  | 38.2 ± 0.1 |  | 36.3 ± 0.1 |  | +0.67 ± 0.11* |  | -1.90 ± 0.13* |
| Morphine |  | L-cysteine |  | NLX |  | 37.6 ± 0.1 |  | 38.2 ± 0.2 |  | 36.3 ± 0.3 |  | +0.68± 0.13* |  | -1.96 ± 0.17* |
| Morphine |  | L-CYSee |  | NLX |  | 37.5 ± 0.1 |  | 37.6 ± 0.1 |  | 37.3 ± 0.1 |  | +0.08 ± 0.09^†^ |  | -0.34 ± 0.08*^,†^ |
| **Body Weight (grams)** | | |  |  |  | **Actual Values** | | | | |  | **ΔChange (grams)** | | |
| **Emulsion** |  | **Infusion** |  | **Injection** |  | **Pre** |  | **+48h** |  | **Post-NLX** |  | **+48h vs Pre** |  | **NLX response** |
| Morphine |  | Vehicle |  | NLX |  | 336 ± 2 |  | 339 ± 2 |  | 328 ± 2 |  | +2.8 ± 1.5 |  | -10.8 ± 1.5* |
| Morphine |  | L-cysteine |  | NLX |  | 335 ± 2 |  | 336 ± 2 |  | 325 ± 2 |  | +1.6 ± 1.1 |  | -11.3 ± 1.6* |
| Morphine |  | L-CYSee |  | NLX |  | 335 ± 2 |  | 337 ± 2 |  | 336 ± 3 |  | +1.4 ± 1.5 |  | -0.8 ± 0.9^†^ |

Responses elicited by the acute injection of NLX (1.5 mg/kg, IP) in rats treated for 36h with a subcutaneous depot of 150 mg/kg morphine and co-infusions of vehicle (20 μL/h, IV) or L-cysteine (20.8 μmol/kg/h, IV) or L-cysteine ethyl ester (L-CYSee, 20.8 μmol/kg/h, IV) for 12 hours starting at 36 hours of morphine administration. There were 9 rats in each group. The data are presented as mean ± SEM. **p* < 0.05, significant responses from Pre. ^†^*p* < 0.05, L-CYSee *versus* vehicle or L-cysteine*.*
